# Direct Infusion Mass Spectrometry to Rapidly Map Metabolic Flux of Substrates Labeled with Stable Isotopes

**DOI:** 10.3390/metabo14050246

**Published:** 2024-04-25

**Authors:** Nils W. F. Meijer, Susan Zwakenberg, Johan Gerrits, Denise Westland, Arif I. Ardisasmita, Sabine A. Fuchs, Nanda M. Verhoeven-Duif, Judith J. M. Jans, Fried J. T. Zwartkruis

**Affiliations:** 1 Department of Genetics, Section Metabolic Diagnostics, University Medical Center Utrecht, Lundlaan 6, 3584 EA Utrecht, The Netherlands; n.w.f.meijer@umcutrecht.nl (N.W.F.M.); n.verhoeven@umcutrecht.nl (N.M.V.-D.); 2 Center for Molecular Medicine, University Medical Center Utrecht, Universiteitsweg 100, 3584 CG Utrecht, The Netherlandsd.westland-2@umcutrecht.nl (D.W.); g.j.t.zwartkruis@umcutrecht.nl (F.J.T.Z.)

**Keywords:** direct infusion–high-resolution mass spectrometry, isotope tracing, glycolysis, TCA cycle, glutaminolysis, organoids, patient material

## Abstract

Direct infusion–high-resolution mass spectrometry (DI-HRMS) allows for rapid profiling of complex mixtures of metabolites in blood, cerebrospinal fluid, tissue samples and cultured cells. Here, we present a DI-HRMS method suitable for the rapid determination of metabolic fluxes of isotopically labeled substrates in cultured cells and organoids. We adapted an automated annotation pipeline by selecting labeled adducts that best represent the majority of ^13^C and/or ^15^N-labeled glycolytic and tricarboxylic acid cycle intermediates as well as a number of their derivatives. Furthermore, valine, leucine and several of their degradation products were included. We show that DI-HRMS can determine anticipated and unanticipated alterations in metabolic fluxes along these pathways that result from the genetic alteration of single metabolic enzymes, including pyruvate dehydrogenase (PDHA1) and glutaminase (GLS). In addition, it can precisely pinpoint metabolic adaptations to the loss of methylmalonyl-CoA mutase in patient-derived liver organoids. Our results highlight the power of DI-HRMS in combination with stable isotopically labeled compounds as an efficient screening method for fluxomics.

## 1. Introduction

The metabolome comprises the complete set of small compounds (metabolites) within a cell or organism and represents the outcome of the interplay of biochemical networks as shaped by enzymes and transporters with its surroundings. The importance of proper metabolic control is evident from numerous inherited inborn metabolic disorders (IMDs), often resulting in devastating disease [1]. In addition, metabolic anomalies contribute to acquired diseases like metabolic syndrome and cancer [2,3]. Metabolomics comprises all studies that focus on the detection and identification of all small molecules within biological samples. Targeted measurements identify and quantify limited numbers of metabolites with high certainty and accuracy. In untargeted approaches, the relative concentration of many more metabolites can simultaneously be detected, but in many instances, isomeric compounds prevent the unambiguous annotation of a given metabolite.

To fully understand the response of a biological system to genetic or environmental alterations, the dynamics of metabolic processes, i.e., metabolic fluxes, should be characterized. Whilst the accumulation of a metabolite suggests increased synthesis, it may equally well be the consequence of decreased processing. Tracing the fate of stable isotopically labeled substrates (e.g., ^15N2^glutamine, ^13C5^glutamine, ^13C6^glucose) over time will uncover the metabolic flux and provide detailed insight in the underlying dynamics of the metabolic network. A major limitation of most current metabolic flux studies is their targeted nature: only a specific subset of metabolites of interest is assessed, depending on pre-existing knowledge of the metabolic network of interest. Current knowledge on metabolic networks, however, is incomplete for most organisms, and targeted approaches may therefore miss the detection of metabolic alterations.

Here, we present a metabolic flux method, based on direct infusion–high-resolution mass spectrometry (DI-HRMS), which we named DI-HRMS-BIT (acronym for DI-HRMS- based isotope tracing). The relatively short analysis time and high technical reproducibility of DI-HRMS are ideal for high-throughput screening and flux analysis. The use of isotope tracing methods based on DI-HRMS has gained attention in recent years. For instance, it has been applied for the quantification of amino acid isotopologues or the evaluation of drug synergies in phenotypic screens [4,5,6,7]. DI-HRMS-BIT takes advantage of the fact that stable isotope tracers result in predictable novel peaks in DI-HRMS spectra with concomitant decreased peak heights of their unlabeled counterparts. Importantly, this holds true for each adduct and consequently allows one to include only those adducts that change for summation of the total intensity of a given metabolite. We demonstrate that DI-HRMS-BIT, in conjunction with the metabolite library developed on the basis of this optimal adduct selection, is suitable to trace the majority of ^13^C-labeled glycolytic, pentose phosphate pathway (PPP) and tricarboxylic acid cycle (TCA) intermediates. In addition, ^15^N and ^13^C-labeled glutamine, aspartic acid, valine and leucine and their catabolic products can be measured. Furthermore, we show that it can be applied to reveal known and unknown metabolic consequences of genetic perturbations in cultured cells and patient-derived organoids.

## 2. Materials and Methods

### 2.1. Cell Culture Conditions

A549 cells were maintained in Roswell Park Memorial Institute (RPMI) 1640 (Thermo Fisher Scientific, Waltham, MA, USA) supplemented with 2 mM of L-glutamine (Sigma Aldrich, Burlington, MA, USA), 10% (*v*/*v*) heat-inactivated fetal bovine serum (FBS) (Thermo Fisher Scientific) and 1% (*v*/*v*) penicillin–streptomycin (P/S) (10,000 U/mL) (Thermo Fisher Scientific). HEK293T cells were cultured in Dulbecco’s Modified Eagle Medium (DMEM) (Thermo Fisher Scientific), 4.5 g/L of glucose, GlutaMAX™, pyruvate, 10% (*v*/*v*) heat-inactivated FBS and 1% (*v*/*v*) P/S. All cell lines were kept at 37 °C in 5% CO_2_ in a humidified incubator and passaged twice a week.

For isotope tracing experiments, cells were seeded in triplicate at a density of 3 × 10^5^ cells per well of a 6-well culture plate (Corning, Corning, NY, USA) and grown until ~70 to 80% confluency. Twenty-four hours prior to the start of the experiment, the medium was replaced with culture medium lacking FBS. The next day, the medium was replaced with FBS-free medium (Table S1) containing the designated isotopically labeled substrate (^13C6^glucose, ^13C5^glutamine, ^15N2^glutamine; Cambridge Isotope Laboratories, Inc., Tewksbury, MA, USA) for the indicated time. For A549::pIND_GLS and A549::pIND_GLS^S482C^, doxycycline was supplemented 3 h prior to the start of the experiment to induce the expression of the respective enzymes. Hereafter, the cells were washed twice with 2 mL of phosphate-buffered saline (PBS) (4 °C). Next, the cells were scraped twice in 0.25 mL of methanol (−80 °C) and transferred to a 1.5 mL Eppendorf tube. Following centrifugation (16,200× *g*, 10 min, 4 °C), the supernatants were transferred to a new 1.5 mL Eppendorf tube (Eppendorf SE, Hamburg, Germany) and stored at −80 °C.

### 2.2. Isotope Tracing in Organoids

For isotope tracing experiments, wild-type and STE177 (homozygous MMUT c.1280 G > T mutation) intrahepatic cholangiocyte organoids were grown in 2D to 80% confluency. To grow the organoids as a 2D culture, approximately 500,000 organoid cells grown in BME were disrupted mechanically into small clumps. The cell suspension was then washed with GF- (without growth factors) and centrifuged for 5 min at 4 °C and 300× *g* twice to remove the basement membrane extract (BME). Cell pellets were suspended in 1 mL of warm culture medium and plated into the well of a 6-well culture plate. After 24 h, 1 mL of medium was added into the well. The medium was changed every 3–4 days.

After the organoids reached 80% confluency, the medium was replaced with Advanced DMEM/F12 medium lacking valine, isoleucine and leucine, supplemented with 2 mM of L-Valine, 2 mM of GlutaMAX, 10 mM of HEPES, 100 U/mL of PenStrep, 2% B27 without vitamin A (Gibco, Grand Island, NY, USA), 10 mM of Nicotinamide (Sigma Aldrich), 1.25 mM of N-Acetylcysteine (Sigma Aldrich), 10 nM of Gastrin (Tocris, Bristol, UK), 50 ng/mL of epidermal growth factor (EGF) (Peprotech, London, UK), 100 ng/mL of FGF10 (Peprotech), 25 ng/mL of hepatocyte growth factor (HGF) (Peprotech), 50 µg/mL of Primocin (InvivoGen, San Diego, CA, USA), 5 μM of A83-01 (Tocris) and 10 μM of Forskolin (Tocris). After 24 h, the medium was replaced again with Advanced DMEM/F12 medium lacking valine, isoleucine and leucine, supplemented with 2 mM of ^13C5^valine for 6 h. Next, the organoids were washed with cold PBS, scraped twice in 0.25 mL of methanol (−80 °C) and transferred to a 1.5 mL Eppendorf tube. Following centrifugation (16,200× *g*, 10 min, 4 °C), the supernatants were transferred to a new 1.5 mL Eppendorf tube and stored at −80 °C.

### 2.3. DI-HRMS-Based Metabolomics

#### 2.3.1. Sample Preparation

Sample cell extracts (70 µL) were diluted with 70 µL of internal standard working solution and 60 µL of 0.3% formic acid (Emsure, Darmstadt, Germany) as described by Haijes et al. [8]. The internal standards included in the working solution are represented in Supplementary Table S2. Next, the samples were filtered using a preconditioned 96-well filter plate (Pall Corporation, Ann Arbor, MI, USA) using a vacuum manifold. The filtrate was collected in an Armadillo high-performance 96-well PCR plate (Thermo Fisher Scientific).

#### 2.3.2. Direct Infusion–High-Resolution Mass Spectrometry

Samples were analyzed by DI-HRMS using a TriVersa NanoMate system (Advion, Ithaca, NY, USA) operated using the Chipsoft software (version 8.3.3, Advion) coupled to a Q Exactive™ Plus hybrid quadrupole-Orbitrap mass spectrometer (Thermo Fisher Scientific) as described previously [8]. Briefly, samples (13 µL) were automatically aspirated into a pipette tip followed by an air gap (2 µL). Next, sample delivery was achieved by engaging the electrospray ionization (ESI) chip with the pipette tip at a nitrogen gas pressure of 0.5 pounds per square inch (PSI) and a spray voltage of 1.6 kilovolts (kV). The system was operated in both negative and positive ion mode over a scan range of 70 to 600 mass to charge ratio (*m*/*z*) and a total run time of 3 min. The scan parameters included a resolution of 140,000, an automatic gain control (AGC) target value of 3 × 10^6^, a maximum injection time of 200 ms, a capillary temperature of 275 °C, an S-Lens RF factor of 70 and a sample tray temperature of 18 °C. In order to improve mass accuracy, mass calibration was performed prior to the measurement. All samples were measured in triplicate.

#### 2.3.3. Data Processing

For DI-HRMS, data acquisition was performed using the Xcalibur software (version 3.0, Thermo Fisher Scientific). Thermo RAW files were converted to mzXML format using ThermoRawFileParser (version 1.1.11, Thermo Fisher Scientific). Next, mzXML files were processed using our customized metabolomics pipeline (source code available at https://github.com/UMCUGenetics/DIMS (accessed on 14 February 2024)). To this end, mass peak annotation was performed by matching the *m*/*z* of the respective peaks to the masses present in our newly developed library in a range of 2 parts per million (Table S3). During the ESI ionization process, adducts are generated; as such, a single metabolite can account for multiple peaks in the mass spectrum representing the addition of the base molecule of interest with different adduct ions (i.e., [M + H]^+^, [M + Na]^+^). The final (summed) mass peak representing the intensity for an individual metabolite was generated by summing the peak intensities of the adducts present within our library both for labeled and corresponding unlabeled metabolites.

### 2.4. Targeted TCA Analysis

#### 2.4.1. Standard and Sample Preparation

Prior to analysis, calibration standards were prepared through dilution and the addition of internal standards in concentration ranges of 0.15 µM to 80 µM. The internal standard working solution included ^2H3^pyruvate, ^2H3^lactate, ^2H4^citrate, ^2H4^2-oxoglutarate, ^2H4^succinate, ^2H4^fumarate and ^2H3^malate (Sigma Aldrich). Next, 20 µL of the internal standard mixture (100 µM) was added to 400 µl of cell extract or 50 µL of calibration standard, evaporated under a flow of nitrogen at 40 °C and reconstituted in a mixture of 25 µL of 0.1% NaOH and 25 µL of 10 mg/mL O-(2,3,4,5,6-pentafluorobenzyl) hydroxylamine (PFBHA) in Milli-Q water. Sample derivatization was performed in a thermomixer at 1000 rpm for 30 min at 20 °C.

#### 2.4.2. Chromatographic Separation

Sample analysis was performed on an Ultimate 3000 UHPLC system (Thermo Fisher Scientific). Chromatographic separation was performed using a Sunshell RP-Aqua column (150 mm × 3 mm i.d., 2.6 µm; ChromaNik Technologies Inc., Osaka, Japan) at 40 °C. To this end, a binary solvent gradient comprising 0.1% formic acid in water (mobile phase A) and 0.1% formic acid in acetonitrile (mobile phase B) was used. The flow rate was 0.6 mL/min with the following gradient elution: isocratic 100% A from 0 to 2.75 min, linear from 100 to 30% A from 2.75 to 3.5 min, isocratic 30% A from 3.5 to 6.5 min, linear from 30 to 100% A from 6.5 to 6.7 min, and isocratic 100% A (initial solvent conditions) from 6.7 to 10 min to equilibrate the column. The column flow was then directed into the MS detector after which the samples were analyzed.

#### 2.4.3. Metabolite Detection

Metabolite detection was performed using a Q Exactive™ HF hybrid quadrupole-Orbitrap mass spectrometer (Thermo Fisher Scientific) with an electrospray ionization source operating in negative mode over a scan range of 70 to 400 *m*/*z*. The scan parameters included a resolution of 240,000, an AGC target value of 1 × 10^6^, a maximum injection time of 200 ms, a capillary (kV) of 4.0 and a capillary temperature of 300 °C, and a factor of 65 was used for the S-lens RF level.

#### 2.4.4. Software/Statistical Analysis

Data acquisition was performed using the Xcalibur software (version 3.0; Thermo Fisher Scientific). Raw data integration was performed using the TraceFinder 4.1 software (Thermo Fisher Scientific).

## 3. Results

### 3.1. Method Development

**Figure 1:**
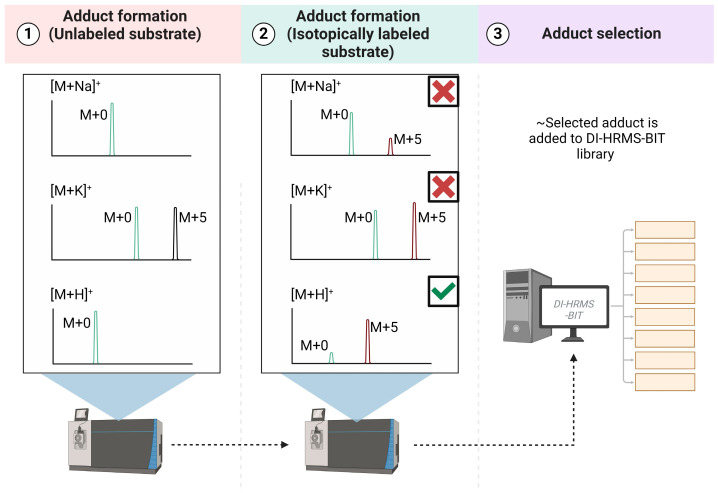
Presence of unlabeled and labeled adduct peaks in spectra derived from cells grown in the presence of unlabeled (1) and isotopically labeled substrates (2). In this example, incorporation of labeled atoms causes a peak shift to M + 5 in the spectrum due to the changes in metabolite mass (2). Comparison between the formation of the labeled adduct peak and subsequent reduction of the unlabeled adduct peak is used as a measure to select those adducts that best represent the metabolite of interest. Adducts were excluded (indicated by the red cross) when either a minor increase was seen in the M + 5 peak (upper spectra) or if a high background peak was present on the location of the labeled peak in unlabeled cell lysates (black peak in middle spectra). Adducts for which a clear increase in the labeled peak and concomitant decrease the unlabeled peak was observed (lower spectra) were included (indicated by the green checkmark) in the library (3). Adapted from “Snapshot Metabolomics” by BioRender.com (2024). Retrieved from https://app.biorender.com/biorender-templates (accessed on 14 February 2024).

The main purpose of this study was to develop a method to capture the dynamics of the metabolome by combining the high-throughput nature of DI-HRMS with the tracing of stable isotopically labeled metabolites. For this, we extended our metabolite library (source code available at https://github.com/UMCUGenetics/DIMS/tree/master/db (accessed on 14 February 2024)) with isotopically labeled ^13^C and/or ^15^N intermediates of the glycolytic pathway, the TCA cycle and side branches including the PPP, the serine biosynthesis route, the malate–aspartate shuttle (MAS) and degradation products of valine and leucine. To this end, downstream products of isotopically labeled substrates and their respective label incorporation were predicted based on The Virtual Metabolic Human database and the KEGG database [9,10,11,12]. For example, we included the expected labeled glycolytic and TCA intermediates as well as products of side branches of the glycolysis including the PPP, the glycerol 3-phosphate shuttle and the serine biosynthetic pathway for ^13C6^glucose (see Supplementary Table S3 for the list of metabolites). Since adducts are formed during the electrospray ionization process, one metabolite can be detected as multiple different adduct ions and thus be represented by different mass peaks in the spectrum. To account for this, adduct lists were created for each labeled version of the metabolites of interest. Here, *m*/*z* values for each labeled adduct (i.e., ^13C4^aspartic acid [M + H]^+^) were defined by summing the *m*/*z* value of the unlabeled adduct (i.e., aspartic acid [M + H]^+^) with the expected increase in the mass to charge ratio caused by the incorporation of four ^13^C-labeled atoms (i.e., 4.01419341344). Adducts included [M + H]^+^, [M + Na]^+^, [M + K]^+^, [M + H + NaCl]^+^, [M + NH4]^+^, [M + 2Na − H]^+^, [M + H + CH3OH]^+^, [M + H + KCl]^+^, [M + NaK − H]^+^ in positive ion mode and [M − H]^−^, [M + Cl]^−^, [M + For]^−^, [M + NaCl − H]^−^, [M + KCl − H]^−^, [M + H2PO4]^−^, [M + HSO4]^−^, [M + Na − 2H]^−^, [M + K − 2H]^−^, [M − 2H]^2−^ and [M + I]^−^ in negative ion mode. To facilitate interpretation, adducts that best represented the labeled metabolites of interest were selected on the basis of measurements with isotopically labeled substrates in cells in pilot experiments. To this end, the spectra of cells grown with unlabeled substrates (i.e., glutamine) were compared to those grown with isotopically labeled substrates (i.e., ^13C5^glutamine). If a metabolite adduct constitutes 100% of the peak that is formed in the presence of an unlabeled substrate, the incorporation of labeled atoms in the presence of an isotopically labeled substrate should cause a peak shift equal to the percentage of label incorporation in that specific metabolite. If no peak shift is observed, this suggests that the particular adduct is not formed or is less readily detected and that the unlabeled peak is likely derived from a different metabolite. As such, an increase in the labeled adduct peak and a concomitant decrease in the unlabeled adduct peak provided validity for the annotation (Figure 1). Only adducts that met both conditions were added to the library. In total, we added 121 labeled compounds corresponding to 41 metabolites. Selected adducts for each metabolite are represented in Supplementary Table S3.

### 3.2. Method Validation

**Figure 2:**
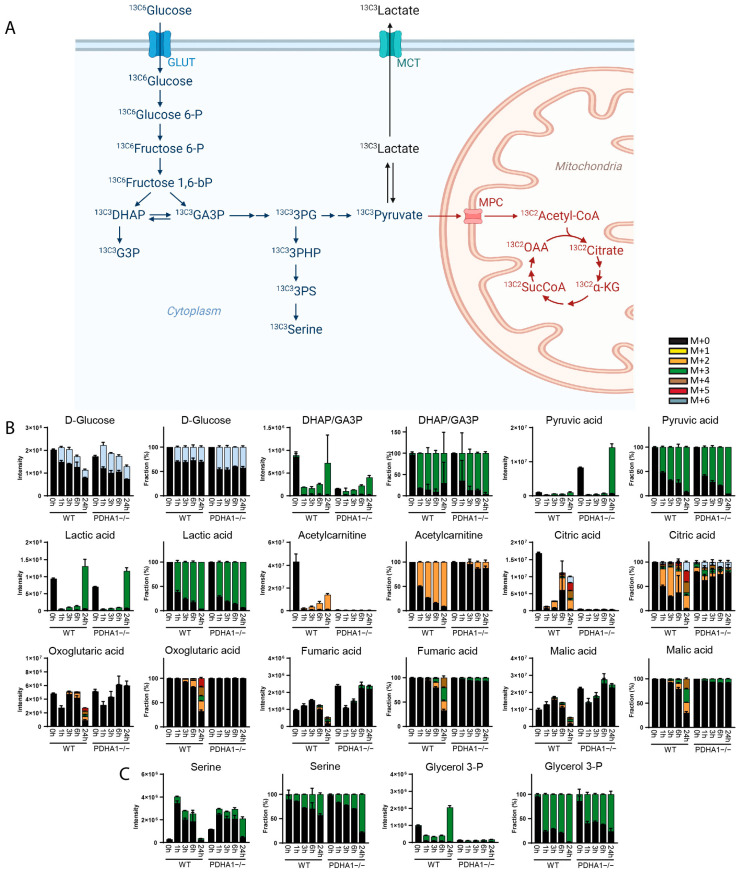
(**A**) Graphical depiction of ^13C6^glucose tracing through glycolysis, TCA cycle and serine biosynthesis. (**B**,**C**) Total (labeled and unlabeled) intensity and fractional enrichment of (**B**) glycolytic and TCA cycle intermediates as well as (**C**) serine and glycerol 3-phosphate after 0 h, 1 h, 3 h, 6 h and 24 h of incubation with ^13C6^glucose in wild-type and PDHA1-deficient HEK293T cells. Isotopologues are described as follows: (M + 0) is unlabeled, (M + 1) contains one ^13^C atom, etc. Isomeric compounds for each component are listed in Table S3. Adapted from “Warburg Effect” by BioRender.com (2023). Retrieved from https://app.biorender.com/biorender-templates (accessed on 14 February 2024).

To confirm that DI-HRMS-BIT could be used to faithfully measure the flux of glucose through glycolysis and the TCA cycle (Figure 2A), wild-type HEK293T cells and a pyruvate dehydrogenase (PDHA1)-deficient counterpart generated with CRISPR-Cas9 were incubated for various timepoints in the presence of ^13C6^glucose. As shown in Figure 2 (and as expected), the labeling of glycolytic compounds started within 60 min and virtually all pyruvate (>95%) was labeled after 24 h [13]. Based on the role of PDH, i.e., the conversion of pyruvate to acetyl-CoA, we anticipated that PDHA1-/- cells would accumulate pyruvate, as these cells cannot use it as a carbon source for the TCA cycle [14,15]. Indeed, kinetic labeling over 24 h led to clearly enhanced ^13C3^pyruvate levels. More strikingly, a very strong decrease in the total amount of unlabeled as well as ^13C2^-labeled citrate was detected by DI-HRMS-BIT in the absence of PDHA1. ^13C2^ and ^13C4^ incorporation into other TCA intermediates was completely absent in PDHA1-negative cells, while about 20 percent of fumaric and malic acid was labeled after 6 h in wild-type cells. Importantly, targeted analysis of the respective cell extracts by LC-HRMS confirmed these results (Figure S2). Label was also incorporated into acetyl-carnitine in wild-type cells, but not PDHA1-deficient cells, reflecting intracellular levels of pyruvate-derived acetyl-CoA. The decrease in unlabeled oxoglutaric acid, malic acid and fumaric acid after 24 h can be explained by reduced glutamine levels in the medium and was seen for both cell lines (Figure S3).

In addition to the expected consequences of PDH deficiency, DI-HRMS-BIT uncovered an enhanced flux of glucose into the serine biosynthesis route that starts from the glycolytic intermediate 3-phosphoglyceric acid. A higher fractional enrichment of ^13C6^glucose-derived ^13C3^-labeled serine was observed after 24 h. In contrast, the formation of labeled glycerol-3-phosphate was decreased in PDHA1-deficient cells, and our measurements confirmed that this compound is continuously and exclusively made as a derivative of glucose (Figure 2C). Low levels of glycerol-3-phosphate may result from the conversion of glycerol-3-phosphate by mitochondrial glycerol 3-phosphate dehydrogenase 2 (GPD2) in the glycerol 3-phosphate shuttle regenerating NAD from NADH. The results shown here demonstrate the capacity of DI-HRMS-BIT to characterize flux through the central carbon metabolism and reveals expected and unexpected changes in metabolites due to a single enzyme deficiency.

**Figure 3:**
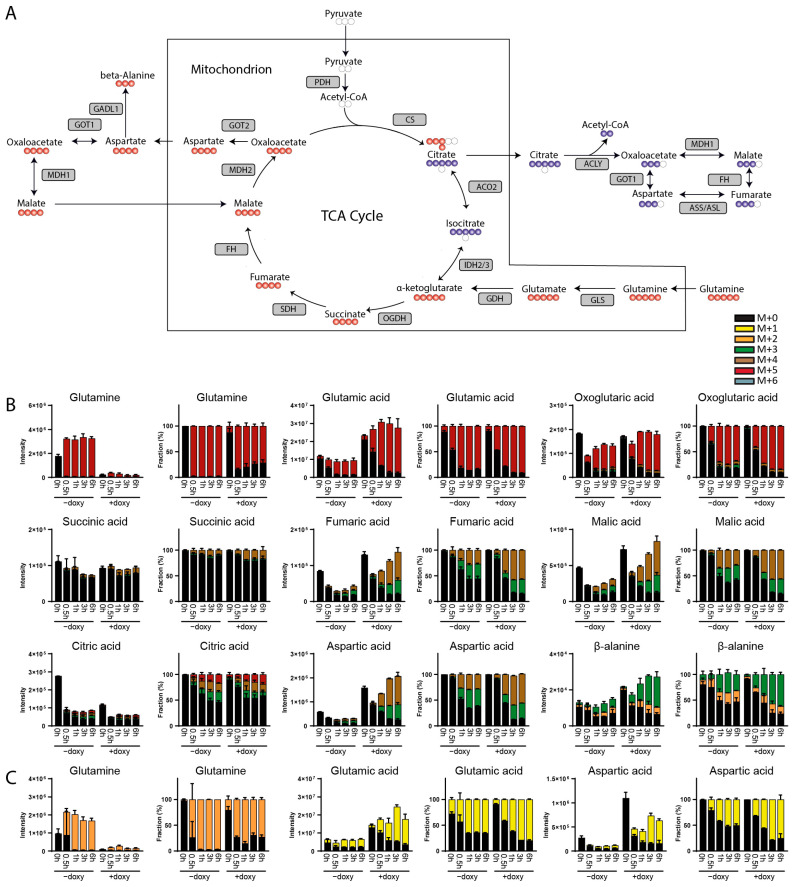
(**A**) Graphical representation of tracing ^13C5^glutamine metabolism through TCA cycle, malate aspartate shuttle and β-alanine synthesis. Red circles (oxidative TCA cycle) and blue circles (reductive carboxylation) indicate ^13^C and white circles indicate ^12^C atoms. (**B**) Total (labeled and unlabeled) intensity and fractional enrichment of TCA cycle and malate aspartate shuttle intermediates as well as β-alanine after 0 h, 0.5 h, 1 h, 3 h and 6 h incubation with ^13C5^glutamine and with/without doxycycline in A549::pIND_GLS^S482C^ cells. (**C**) Total (labeled and unlabeled) intensity and fractional enrichment of glutamine, glutamic acid and aspartic acid after 0 h, 0.5 h, 1 h, 3 h and 6 h incubation with ^15N2^glutamine and with/without doxycycline in A549::pIND_GLS^S482C^ cells. Isotopologues are described as follows: (M + 0) is unlabeled, (M + 1) contains one ^13^C or ^15^N atom, etc. Isomeric compounds for each component are listed in Table S3.

An important application of isotope tracer analysis is to determine which carbon sources are used for energy production and the generation of biomass. For A549 cells, it has been established that biomass is mostly acquired from amino acids, while glucose-derived carbon is largely excreted as lactic acid [16]. A549 cells compensate for the low influx of pyruvate into the TCA cycle by using glutamine for the generation of ATP via the TCA cycle (Figure 3A) [17]. We therefore used two variants of this cell line to follow the fate of ^13C5^glutamine. In A549::pIND_GLS cells, overexpression of wild-type GLS is under the control of the doxycycline-responsive promoter, while in A549::pIND_GLS^S482C^ cells, a hyperactive and pathogenic variant can be induced [18]. A rapid and massive conversion of ^13C5^glutamine to ^13C5^glutamate and ^13C5^oxoglutarate was seen in the absence of doxycycline in both cell lines (Figure 3B and Figure S4). Oxoglutarate entered the TCA cycle, resulting in approximately 25% of ^13C4^-labeled fumarate and malic acid after 6 h. Additionally, a slightly lower fraction of ^13C3^-labeled fumaric and malic acid was generated via reductive carboxylation (Figure 3B). These findings are well in line with the metabolic flux model for A549 cells that has been established based on the use of different labeled tracers [19]. In contrast to that model, DI-HRMS-BIT revealed a considerable formation of aspartate from malate. Overexpression of wild-type GLS with doxycycline had little effect on the pattern of incorporation of labeled carbons (Figure S4). In contrast, induced expression of GLS^S482C^ resulted in a dramatic decrease in glutamine levels and a strong increase in glutamate. The fractional enrichment of ^13C5^glutamate was similar to that in the absence of GLS^S482C^, showing that increased glutamate levels were not caused by diminished consumption (Figure 3B). Consistent with the notion that glutaminolysis functions to replenish TCA cycle intermediates, we observed an increase in the total levels of fumaric acid and malic acid upon GLS^S482C^ expression [20,21]. With regard to modulation in TCA pathway orchestration by GLS^S482C^, an increase in the relative contribution to the oxidative versus reductive part of the TCA cycle (increased ratio of M + 4 to M + 3 fumarate and malate) was seen (Figure 3B). Moreover, a significant increase in the total as well as the ^13C4^-labeled fraction of aspartic acid was found in the presence of the hyperactive GLS variant. Here, limited amounts of glucose-derived acetyl-CoA likely directs oxaloacetate towards aspartate via mitochondrial glutamic oxaloacetic transaminase (GOT2) to maintain TCA cycle flow for subsequent ATP production. DI-HRMS-BIT also pointed to the formation of a compound corresponding in mass with ^13C3^alanine (Figure 3B). Given the absence of labeled pyruvate and based on the adducts observed, we assigned this peak to ^13C3^beta-alanine synthesized via the decarboxylation of aspartate by GADL1 (Figure 3A). To further evaluate the applicability of DI-HRMS-BIT, ^15N2^glutamine was used as a tracer in A549::pIND_GLS^S482C^ cells. In agreement with the outcome of ^13C5^glutamine tracing, strongly decreased ^15N2^glutamine and enhanced ^15N1^glutamate and ^15N1^aspartate levels were observed in cells that expressed GLS^S482C^ (Figure 3C). In summary, ^13C5^glutamine and ^15N2^glutamine tracing in A549 cells using DI-HRMS-BIT reproduced previous observations in this cell line and efficiently captured the consequences of hyperactive GLS.

**Figure 4:**
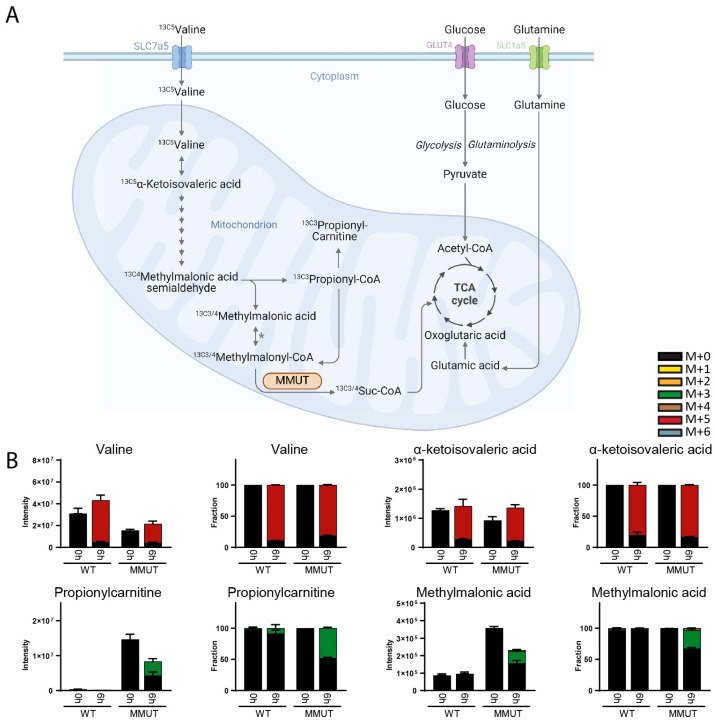
(**A**) Graphical representation of tracing ^13C5^valine metabolism through the valine breakdown pathway and TCA cycle. (**B**) Total (labeled and unlabeled) intensity and fractional enrichment of valine, α-ketoisovaleric acid, propionylcarnitine and methylmalonic acid after 0 h and 6 h incubation with ^13C5^valine in organoids derived from healthy individuals and patients with a loss-of-function mutation (STE177, homozygous MMUT c.1280 G > T mutation) in methylmalonyl-CoA mutase (MMUT). Isotopologues are described as follows: (M + 0) is unlabeled, (M + 1) contains one ^13^C atom, etc. Isomeric compounds for each component are listed in Table S3. Adapted from “Overview of BCAA Catabolic Enzymes” by BioRender.com (2023). Retrieved from https://app.biorender.com/biorender-templates (accessed on 14 February 2024). * The enzyme responsible for the conversion of methylmalonyl-CoA to methylmalonic acid is not identified and listed in KEGG as 3.1.2.17.

One of our intended purposes of isotope tracing with DI-HRMS-BIT is to map metabolic anomalies in patient-derived material. Unlike the cell lines described above with complete loss-of-function or gain-of-function mutations, genetic alterations in patient material may only partially affect enzymatic activity, resulting in more subtle metabolic changes or involving metabolites with low abundance. Therefore, we examined liver organoids derived from healthy individuals and patients with an MMUT^G426D^ mutation (clone STE177, homozygous MMUT c.1280 G > T), localizing to the dimerization domain in methylmalonyl-CoA mutase (MMUT) and classified as a mut- mutation, meaning it has residual activity or is vitamin B_12_-responsive [22]. Kinetic labeling with ^13C5^valine over 6 h uncovered labeling of the majority of valine and alpha-ketoisovalerate (Figure 4A,B). Although valine was somewhat decreased in mutant organoids, propionyl carnitine, frequently used as a biomarker for MMA, was increased, with fifty percent originating from labeled valine. The MMUT isomerization products methylmalonyl-CoA and succinyl-CoA were not resolved, but the concentration and labeling of methylmalonic acid (upstream of MMUT) were increased in mutant organoids, demonstrating severely diminished enzymatic activity of the MMUT^G426D^ mutation (Figure 4B).

## 4. Discussion

Here, we describe a high-throughput method to perform metabolic flux analysis using DI-HRMS. This method is suitable for determining the fate of stable isotopically labeled metabolites such as glucose and glutamine derivatives through central carbon metabolism over time. In addition, degradative pathways of amino acids like valine and leucine can be monitored. A limitation of DI-HRMS is that the introduction of multiple compounds into the ionization source may result in ion suppression or enhancement. In this regard, the quantification of metabolic fluxes by targeted approaches may yield more accurate quantitative results and can be optimized for the detection of certain metabolites that cannot be discriminated by DI-HRMS-BIT. However, we think that this novel method has several advantages. Firstly, this universal platform can be used to determine the conversion of various stable isotopically labeled metabolites with high throughput. Second, the nature of DI-HRMS measurements provides a broader scope of cellular metabolism as profiling labeled compounds is seen in the context of many non-labeled metabolites. Finally, the strict selection of adducts as described below assures the correct annotation of labeled and unlabeled metabolites. Of course, this method also has limitations: the acquired library may have matrix-specific characteristics, starts with knowledge about previously described metabolic pathways, and requires experimental data. However, it does not require complex computational approaches that analyze mass spectra in a more unbiased manner [5,6,23,24]. We consider our method complementary to such computational approaches.

Using genome-edited and virally transduced cell lines, we demonstrate that DI-HRMS-BIT readily detects expected metabolic consequences like a block in pyruvate consumption via the TCA cycle in PDHA1-defective HEK293T cells. Furthermore, unexpected changes, including decreased production of glycerol 3-phosphate in PDHA1 knockout cells, were readily observed. The inspection of the DI-HRMS spectra of cell extracts with labeled metabolites mostly revealed the expected appearance of novel peaks representing labeled compounds and the simultaneous disappearance of unlabeled counterparts. When this was not the case, possibly because certain adducts are not detected or are less readily formed, we removed the corresponding adduct mass for the detection of the unlabeled equivalent. In fact, this improves the accuracy of annotation for unlabeled compounds as seen by a reduction in noise. A future extension of the database with labeled masses of stable isotope tracers should be automated using this approach. In addition, in the future, automated recognition of novel peaks might reveal previously unrecognized transformations of metabolites and thus extend our knowledge of metabolic pathways.

One of our goals of DI-HRMS-BIT is to identify metabolic anomalies in IMDs. Nowadays, most analyses are performed in patient-derived body fluids (like blood and urine) or fibroblasts. Options to grow patient material, either from induced pluripotent cells (iPSCs) or from adult stem cells (like the liver organoids in our studies), allow metabolomic analyses in organ- and patient-specific models. The findings in these models may be less predictable and more subtle than those we found in our genetically engineered cell lines. To demonstrate that DI-HRMS-BIT can reveal more subtle metabolic abnormalities, ^13C5^valine tracing was performed in liver organoids from MMA patients, and the outcomes were in excellent agreement with the genetic defect in MMUT. Our approach may also be valuable in clarifying genetic findings of unknown significance.

## 5. Conclusions

Here, we describe the development of a novel method in which improved annotation on the basis of the adduct selection of metabolites allows for robust isotope tracing. Validation in cellular and organoid models confirmed an improved assignment of mass peaks. Therefore, DI-HRMS-BIT is a valuable and complementary addition to other metabolomics methods and may be used for the investigation of metabolic pathways involved in central carbon and amino acid metabolism.

## 6. Limitations of This Study

We envision two ways of applying the outcomes of this study. First, researchers may directly run the pipeline including this database of labeled metabolites. Second, the line of reasoning as presented in Figure 1 may help researchers to adapt their own in-house pipeline. However, a limitation of this approach is that adducts were selected based on experimental data in cultured cells. As a result, selected adducts may perform poorly when different biological matrices are analyzed. Conversely, adducts that were discarded may perform well in other biological matrices.

## Data Availability

The data presented in this study are available on request from the corresponding author.

## Supplementary Materials

The following supporting information can be downloaded at https://www.mdpi.com/article/10.3390/metabo14050246/s1: Supplementary materials and methods including the generation of a genome-edited cell line by CRISPR-Cas9; the generation of GLS-overexpressing A549 cells; and organoid culture. Table S1: Medium used for isotope tracing experiments; Table S2: Composition of the internal standard working solution; Table S3: DI-HRMS-BIT library depicting the assigned HMDB names and corresponding adducts with *m*/*z* values used to annotate mass peaks derived from DI-HRMS in positive and negative ion mode; Figure S1: Western blot analysis of total lysates from wild-type and PDHA1-/- HEK293T cells; Figure S2: Targeted profiling of indicated metabolites in wild-type and PDHA1-deficient HEK293T cells cultured in the presence of ^13C6^glucose for 0 h, 1 h, 3 h, 6 h and 24 h; Figure S3: Total (labeled and unlabeled) intensity and fractional enrichment of glutamine in medium after 0 h, 1 h, 3 h, 6 h and 24 h incubation with ^13C6^glucose in wild-type and PDHA1-deficient HEK293T cells; Figure S4: Metabolic consequences of overexpression of wild-type GLS [18,25,26,27,28].
