# Supplementary material for: Direct Infusion Mass Spectrometry to Rapidly Map Metabolic Flux of Substrates Labeled with Stable Isotopes
Source: Metabolites. 2024 Apr 25;14(5):246. doi: 10.3390/metabo14050246 (PMC11122925; doi:10.3390/metabo14050246)
Supplement: Supplementary file 1 [file metabolites-14-00246-s001.zip › metabolites-2934871-supplementary.pdf]

Supplementary information

Supplementary Materials and Methods

Generation of genome-edited cell line by CRISPR-Cas9

In order to disrupt Pyruvate Dehydrogenase E1 Subunit Alpha 1 (PDHA1), CRISPR-Cas9 mediated genome editing was applied as previously described [25]. In brief, Cas9 and single guide RNAs (caccGATGCAGACTGTACGCCGAA and aaacTTCGGCGTACAGTCTGCATC) were simultaneously expressed using the PX458 plasmid by transient transfection using Xtremegene [26]. Transfected cells were FACS sorted and single cells were expanded. Analysis of resulting clones was done by western blotting using the following antibodies: Pyruvate Dehydrogenase (3205S; Cell Signaling Technology), glyceraldehyde 3-phosphate dehydrogenase (GAPDH) (MAB374; Merck Millipore) (Figure S1).

Generation of GLS over-expressing A549 cells

cDNAs of glutaminase (GLS) or GLS^{S482C} (UniProt O94925, isoform 1, also known as GLS1 or KGA) were amplified by polymerase chain reaction (PCR) using the pcDNA5/FRT/TO vector as previously described and the primers aaaaaagcaggcttccggggatcccGCGGCATGATGCGGCTGCGAGGCTCGGGGA and aaagctgggtcgcggccgcATTACAACAATCCATCAAGATTCTT [18]. The cDNAs were cloned into pEntry and shuttled to pInducer20 using standard gateway cloning [27]. A549 cells were infected with lentiviruses made with the GLS-encoding pInducer20 constructs and selected with G418.

Organoid culture

The study was approved by the responsible local ethics committees (Institutional Review Board of the University Medical Center Utrecht (STEM: 10-402/K; TcBio 14-008; Metabolic Biobank: 19-489, active since 05-06-2020). All biopsies were used after written informed consent. Intrahepatic cholangiocyte organoids were established and cultured as described previously [28]. Liver biopsies were cut into small pieces and digested using 10 mg/ml Collagenase D (Sigma Aldrich) in Hanks' Balanced Salt Solution (HyClone, Thermo Fisher Scientific) for 20 min at 37 °C. Digested samples were then diluted and washed with cold GF-(without growth factors) (Advanced DMEM/F12 (Gibco) supplemented with 2 mM GlutaMAX (Gibco), 10 mM HEPES (Gibco), 100 U/ml Pen-Strep (Gibco) and centrifuged for 5 min, 4 °C at 300 g. The cell pellet was plated in basement membrane extract (BME) (Bio-techne) and culture medium was added. Culture medium was based on Advanced DMEM/F12 supplemented with 2 mM GlutaMAX, 10 mM HEPES, 100 U/ml PenStrep, 2 % B27 without vitamin A (Gibco), 10 mM Nicotinamide (Sigma Aldrich), 1.25 mM N-Acetylcysteine (Sigma Aldrich), 10 % R-Spondin 1 (RSPO1) conditioned media (homemade), 10 nM Gastrin (Tocris), 50 ng/ml epidermal growth factor (EGF) (Peprotech), 100 ng/ml FGF10 (Peprotech), 25 ng/ml hepatocyte growth factor (HGF) (Peprotech), 50 µg/ml Primocin (InvivoGen), 5 µM A83-01 (Tocris), and 10 µM Forskolin (Tocris). For the first 3 days in culture, the medium was supplemented with 30 % Wnt conditioned media (homemade), 25 ng/ml Noggin (Peprotech), and human embryonic stem (hES) cell cloning recovery solution (Stemgent). The medium was changed every 3–4 days and organoids were passaged 1:4–1:8 each week.

Supplementary tables

Table S1. Medium used for isotope tracing experiments

**Cell line**	**Medium**
HEK293T-WT<br><br>HEK293T-PDHA1-/-	DMEM, no glucose and no sodium-pyruvate (ThermoFisher Scientific) 1 % P/S (v/v) and 25 mM D-Glucose (^{13C6}glucose, 99%, Cambridge Isotope Laboratories, Inc.)

A549-WT<br><br>A549::pIND_GLS	RPMI 1640 medium, no glutamine (ThermoFisher Scientific), 1 % P/S (v/v) and 2 mM ^{13C5}Glutamine (99%, Cambridge Isotope Laboratories, Inc.)
A549::pIND_GLS^{S482C}	RPMI 1640 medium, no glutamine (ThermoFisher Scientific), 1 % P/S (v/v) and 2 mM ^{15N2}Glutamine (98%, Cambridge Isotope Laboratories, Inc.)

Table S2. Composition of the internal standard working solution

**Standard**	**Concentration**
$^{15N2-13C}$glycine	100 µM (Cambridge Isotope Laboratories, Buchem b.v., Apeldoorn, The Netherlands)
^{2H4}alanine, ^{2H3}leucine, ^{2H3}methionine, ^{13C6}phenylalanine, ^{13C6}tyrosine, ^{2H3}aspartate, ^{2H3}glutamate, ^{2H2}ornithine, ^{2H2}citrulline and $^{2H4-13C}$arginine	20 µM (Cambridge Isotope Laboratories, Buchem b.v., Apeldoorn, The Netherlands)
^{2H9}carnitine	6.08 µM (Cambridge Isotope Laboratories, Buchem b.v., Apeldoorn, The Netherlands)
^{2H3}acetylcarnitine	1.52 µM (Cambridge Isotope Laboratories, Buchem b.v., Apeldoorn, The Netherlands)
^{2H3}palmitoylcarnitine	0.608 µM (Cambridge Isotope Laboratories, Buchem b.v., Apeldoorn, The Netherlands)
^{2H3}propionylcarnitine, ^{2H3}butyrylcarnitine, ^{2H9}isovalerylcarnitine, ^{2H3}octanoylcarnitine, ^{2H9}myristoylcarnitine	0.304 µM (Cambridge Isotope Laboratories, Buchem b.v., Apeldoorn, The Netherlands)

Table S3. DI-HRMS-BIT library depicting the assigned HMDB names and corresponding adducts with m/z values used to annotate mass peaks derived from DI-HRMS in positive and negative ion modus.

**Assigned HMDB name**	**[Isotopologue]**	**theoretical m/z**
**Glucose**		
**D-Glucose**;D-Galactose;D-Mannose;Myoinositol;3-Deoxyarabinohexonic acid;Beta-D-Glucose;D-	$[M+H]^+$<br>$[M+Na]^+$<br>$[M+K]^+$	181.070665<br>203.052607<br>219.026547

Fructose;Allose;L-Sorbose;Alpha-D-Glucose;D-Tagatose;Beta-D-Galactose;Scyllitol;L-Gulose;L-Galactose	[M-H]$^-$<br>[M+Cl]$^-$	179.056112<br>215.032789
^{13C6}Glucose	[M+H]$^+$<br>[M+Na]$^+$<br>[M+K]$^+$<br>[M-H]$^-$<br>[M+Cl]$^-$	187.090794<br>209.072736<br>225.046676<br>185.076241<br>221.052918
**Glucose 6-phosphate/ Fructose 6-phosphate**		
**Glucose 6-phosphate;Fructose 6-phosphate**;Myo-inositol 1-phosphate;Galactose 1-phosphate;Dolichyl phosphate D-mannose;Fructose 1-phosphate;Mannose 6-phosphate;D-Myo-inositol 4-phosphate;Glucose 1-phosphate;Inositol phosphate;Beta-D-Glucose 6-phosphate;Beta-D-Fructose 6-phosphate;D-Tagatose 1-phosphate;D-Mannose 1-phosphate;Sorbose 1-phosphate;Beta-D-Fructose 2-phosphate;1D-myo-Inositol 3-phosphate;D-Tagatose 6-phosphate;D-fructose 1-phosphate	[M-H]$^-$	259.022442
^{13C6}Glucose 6-phosphate;^{13C6}Fructose 6-phosphate	[M-H]$^-$	265.042571
**Fructose 1,6-bisphosphate**		
**Fructose 1,6-bisphosphate**;"1D-Myo-inositol 1,4-bisphosphate";"D-Fructose 2,6-bisphosphate";"Alpha-D-Glucose 1,6-bisphosphate";"1D-Myo-inositol 1,3-bisphosphate";"1D-Myo-inositol 3,4-bisphosphate";"D-Tagatose 1,6-bisphosphate";"D-Mannose 1,6-bisphosphate";"beta-D-Fructose 1,6-bisphosphate"	[M-H]$^-$	338.988773
^{13C6}Fructose 1,6-bisphosphate	[M-H]$^-$	345.008902
**Glyceraldehyde 3-phosphate/ Dihydroxyacetone phosphate**		
**D-Glyceraldehyde 3-phosphate;Dihydroxyacetone phosphate**	[M-H]$^-$	168.990748
^{13C6}D-Glyceraldehyde 3-phosphate;Dihydroxyacetone phosphate	[M-H]$^-$	172.000812
**Glyceric acid 1,3-biphosphate**		
**Glyceric acid 1,3-biphosphate**;2,3-Diphosphoglyceric acid	[M-H]$^-$	264.951993
^{13C3}Glyceric acid 1,3-biphosphate	[M-H]$^-$	267.962058
**2-phosphoglycerate/ 3-phosphoglycerate**		
**2-Phosphoglyceric acid;3-Phosphoglyceric acid**;2-Phospho-D-glyceric acid;(2R)-2-Hydroxy-3-(phosphonatooxy)propanoate	[M-H]$^-$	184.985663
^{13C3}2-Phosphoglyceric acid;3-Phosphoglyceric acid	[M-H]$^-$	187.995727
**Phosphoenolpyruvic acid**		
Phosphoenolpyruvic acid	[M-H]$^-$	166.975098
^{13C3}Phosphoenolpyruvic acid	[M-H]$^-$	169.985163
**Pyruvate**		
**Pyruvic acid**;Malonic semialdehyde	[M-H]$^-$	87.008768
^{13C3}Pyruvic acid	[M-H]$^-$	90.018833
**Lactate**		

**L-Lactic acid**;Hydroxypropionic acid;Glyceraldehyde;D-Lactic acid;Dihydroxyacetone;Methoxyacetic acid	[M-H]$^-$	89.024418
^{13C3}L-Lactic acid	[M-H]$^-$	92.034483
**Glycerol 3-phosphate**		
**Glycerol 3-phosphate**;Beta-Glycerophosphoric acid	[M-H]$^-$	171.006398
^{13C3}Glycerol 3-phosphate	[M-H]$^-$	174.016463
**Phosphohydroxypyruvate**		
**Phosphohydroxypyruvic acid**;3-Phosphonatooxypyruvate	[M-H]$^-$	182.970013
^{13C3}Phosphohydroxypyruvic acid	[M-H]$^-$	185.980077
**Phosphoserine**		
**Phosphoserine**	[M+H]$^+$<br>[M-H]$^-$	186.016110<br>184.001647
^{13C3}Phosphoserine	[M+H]$^+$<br>[M-H]$^-$	189.026264<br>187.011712
**Serine**		
**Serine**	[M+H]$^+$<br>[M+Na]$^+$<br>[M+K]$^+$<br>[M-H]$^-$	106.049870<br>128.031812<br>144.005752<br>104.035317
^{13C3}Serine	[M+H]$^+$<br>[M+Na]$^+$<br>[M+K]$^+$<br>[M-H]$^-$	109.059934<br>131.041877<br>147.015817<br>107.045381
**L-Acetylcarnitine**		
**L-Acetylcarnitine**	[M+H]$^+$	204.123034
^{13C2}L-Acetylcarnitine	[M+H]$^+$	206.129744
**Citrate**		
**Citric acid**;Isocitric acid;D-threo-Isocitric acid;Diketogulonic acid;"2,3-Diketo-L-gulonate";"(1R,2R)-Isocitric acid";"D-Glucaro-1,4-lactone"	[M-H]$^-$	191.019726
^{13C2}Citrate	[M-H]$^-$	193.026436
^{13C3}Citrate	[M-H]$^-$	194.029791
^{13C4}Citrate	[M-H]$^-$	195.033146
^{13C5}Citrate	[M-H]$^-$	196.036500
^{13C6}Citrate	[M-H]$^-$	197.039855
**2-oxoglutarate**		
**2-oxoglutarate**	[M-H]$^-$	145.014248
^{13C2}2-oxoglutarate	[M-H]$^-$	147.020957
^{13C3}2-oxoglutarate	[M-H]$^-$	148.024311
^{13C4}2-oxoglutarate	[M-H]$^-$	149.027666
^{13C5}2-oxoglutarate	[M-H]$^-$	150.031021
**Succinate**		
**Succinate**;Methylmalonic acid;Erythrono-1,4-lactone;Threonolactone	[M-H]$^-$	117.019332
^{13C2}Succinate	[M-H]$^-$	119.026042
^{13C3}Succinate	[M-H]$^-$	120.029397
^{13C4}Succinate	[M-H]$^-$	121.032752
**Fumarate**		

**Fumarate**;Maleic acid	[M-H]-	115.003682
13C2Fumarate	[M-H]-	117.009858
13C3Fumarate	[M-H]-	118.013918
13C4Fumarate	[M-H]-	119.017375
**Malate**		
Malate	[M-H]-	133.014247
13C2Malate	[M-H]-	135.020957
13C3Malate	[M-H]-	136.024311
13C4Malate	[M-H]-	137.027666
**Aspartate**		
**L-Aspartate**;D-Aspartate;Iminodiacetic acid	[M+Na]+<br>[M-H]-<br>[M+Cl]-	156.026727<br>132.030231<br>168.006909
13C2L-Aspartate	[M+Na]+<br>[M-H]-<br>[M+Cl]-	158.033436<br>134.036941<br>170.013618
13C3L-Aspartate	[M+Na]+<br>[M-H]-<br>[M+Cl]-	159.036791<br>135.040296<br>171.016973
13C4L-Aspartate	[M+Na]+<br>[M-H]-<br>[M+Cl]-	160.040146<br>136.043651<br>172.020328
15N1L-Aspartate	[M+Na]+<br>[M-H]-<br>[M+Cl]-	157.023762<br>133.027266<br>169.003944
**Glutamine**		
**L-Glutamine**;Ureidoisobutyric acid;D-Glutamine;Alanylglycine	[M+H]+<br>[M+Na]+<br>[M+K]+<br>[M-H]-<br>[M+Cl]-	147.076419<br>169.058361<br>185.032301<br>145.0618657<br>181.0385432
13C2L-Glutamine	[M+H]+<br>[M+Na]+<br>[M+K]+<br>[M-H]-<br>[M+Cl]-	149.0831287<br>171.0650707<br>187.0390107<br>147.0685754<br>183.0452529
13C3L-Glutamine	[M+H]+<br>[M+Na]+<br>[M+K]+<br>[M-H]-<br>[M+Cl]-	150.0864835<br>172.0684255<br>188.0423655<br>148.0719302<br>184.0486077
13C4L-Glutamine	[M+H]+<br>[M+Na]+<br>[M+K]+<br>[M-H]-<br>[M+Cl]-	151.0898384<br>173.0717804<br>189.0457204<br>149.0752851<br>185.0519626
13C5L-Glutamine	[M+H]+<br>[M+Na]+<br>[M+K]+<br>[M-H]-	152.0931932<br>174.0751352<br>190.0490752<br>150.0786399

	[M+Cl]-	186.0553174
^{15N1}L-Glutamine	[M+H]+<br>[M+Na]+<br>[M+K]+<br>[M-H]-<br>[M+Cl]-	148.0734539<br>170.0553959<br>186.0293359<br>146.0589006<br>182.0355781
^{15N2}L-Glutamine	[M+H]+<br>[M+Na]+<br>[M+K]+<br>[M-H]-<br>[M+Cl]-	149.0704888<br>171.0524308<br>187.0263708<br>147.0559355<br>183.0326130
**Glutamate**		
**L-Glutamic acid**;N-Methyl-D-aspartic acid;N-Acetylserine;D-Glutamic acid;L-4-Hydroxyglutamate semialdehyde;DL-Glutamate	[M+H]+<br>[M+Na]+<br>[M+K]+<br>[M-H]-<br>[M+Cl]-	148.060434<br>170.042377<br>186.016317<br>146.045881<br>182.022559
^{13C2}L-Glutamic acid	[M+H]+<br>[M+Na]+<br>[M+K]+<br>[M-H]-<br>[M+Cl]-	150.067144<br>172.049086<br>188.023026<br>148.052591<br>184.029268
^{13C3}L-Glutamic acid	[M+H]+<br>[M+Na]+<br>[M+K]+<br>[M-H]-<br>[M+Cl]-	151.070499<br>173.052441<br>189.026381<br>149.055946<br>185.032623
^{13C4}L-Glutamic acid	[M+H]+<br>[M+Na]+<br>[M+K]+<br>[M-H]-<br>[M+Cl]-	152.073854<br>174.055796<br>190.029736<br>150.059301<br>186.035978
^{13C5}L-Glutamic acid	[M+H]+<br>[M+Na]+<br>[M+K]+<br>[M-H]-<br>[M+Cl]-	153.077208<br>175.059151<br>191.033091<br>151.062656<br>187.039333
^{15N1}L-Glutamic acid	[M+H]+<br>[M+Na]+<br>[M+K]+<br>[M-H]-<br>[M+Cl]-	149.057469<br>171.039412<br>187.013352<br>147.042916<br>183.019594
**Beta-alanine/ L-Alanine**		
**Beta-alanine;L-Alanine;**Sarcosine;D-Alanine;Ethyl carbamate	[M-H]-	88.040402
^{13C2}Beta-Alanine/^{13C2}L-Alanine	[M-H]-	90.047112
^{13C3}Beta-Alanine/^{13C3}L-Alanine	[M-H]-	91.050467
^{15N1}Beta-Alanine/^{15N1}L-Alanine	[M-H]-	89.037437
**L-Asparagine**		
**L-Asparagine;**Ureidopropionic acid; Glycyl-glycine;N-Carbamoylsarcosine;D-Asparagine	[M+H]+<br>[M+Na]+	133.060769<br>155.042711

	[M+K]+<br>[M-H]-<br>[M+Cl]-	171.016651<br>131.046216<br>167.022893
13C2L-Asparagine	[M+H]+<br>[M+Na]+<br>[M+K]+<br>[M-H]-<br>[M+Cl]-	135.067478<br>157.049421<br>173.023361<br>133.052925<br>169.029603
13C3L-Asparagine	[M+H]+<br>[M+Na]+<br>[M+K]+<br>[M-H]-<br>[M+Cl]-	136.070833<br>158.052776<br>174.026716<br>134.056280<br>170.032958
13C4L-Asparagine	[M+H]+<br>[M+Na]+<br>[M+K]+<br>[M-H]-<br>[M+Cl]-	137.074188<br>159.056130<br>175.030070<br>135.059635<br>171.036312
15N1L-Asparagine	[M+H]+<br>[M+Na]+<br>[M+K]+<br>[M-H]-<br>[M+Cl]-	134.057803<br>156.039746<br>172.013686<br>132.043251<br>168.019928
15N2L-Asparagine	[M+H]+<br>[M+Na]+<br>[M+K]+<br>[M-H]-<br>[M+Cl]-	135.054838<br>157.036781<br>173.010721<br>133.040286<br>169.016963
**Proline**		
**L-Proline**;D-Proline;Acetamidopropanal;<br>4-Amino-2-methylenebutanoic acid;Pterolactam;	[M+H]+<br>[M+Na]+<br>[M+K]+	116.070605<br>138.052548<br>154.026488
13C5L-Proline	[M+H]+<br>[M+Na]+<br>[M+K]+	121.087379<br>143.069322<br>159.043262
15N1L-Proline	[M+H]+<br>[M+Na]+<br>[M+K]+	117.067640<br>139.049582<br>155.023522
**Glycine**		
**Glycine**;Acetohydroxamic Acid;Ethyl nitrite	[M+H]+<br>[M+Na]+	76.039305<br>98.021247
13C2Glycine	[M+H]+<br>[M+Na]+	78.046015<br>100.027957
**Ornithine**		
**Ornithine**;D-Ornithine	[M+H]+	133.097154
13C1Ornithine	[M+H]+	134.100509
13C5Ornithine	[M+H]+	138.113928
**Leucine**		
**L-Leucine;**L-Isoleucine;L-Alloisoleucine; L-<br>Norleucine;Aminocaproic acid;Beta-Leucine;D-Leucine;3-	[M+H]+<br>[M+Na]+<br>[M+K]+	132.101905<br>154.083848<br>170.057788

Aminocaproic acid;erythro-Isoleucine;6-Deoxyfagomine;N-(2-Hydroxyethyl)-morpholine;	[M-H]$^-$<br>[M+Cl]$^-$	130.087352<br>166.064030
^{13C6}L-Leucine	[M+H]$^+$<br>[M+Na]$^+$<br>[M+K]$^+$<br>[M-H]$^-$<br>[M+Cl]$^-$	138.122034<br>160.103977<br>176.077917<br>136.107481<br>172.084159
^{15N1}L-Leucine	[M+H]$^+$<br>[M+Na]$^+$<br>[M+K]$^+$<br>[M-H]$^-$<br>[M+Cl]$^-$	138.122034<br>160.103977<br>176.077917<br>136.107481<br>172.084159
**Ketoleucine**		
**Ketoleucine;**2-Methyl-3-ketovaleric acid;3-Methyl-2-oxovaleric acid; 2-Ketohexanoic acid;Mevalonolactone;3-Oxohexanoic acid;Adipate semialdehyde;"5-Ethoxy-4,5-dihydro-2(3H)furanone";Ethyl acetoacetate;Ethyl 3-oxobutanoate;Sherry lactone;"(4S,6S)-3,4,5,6-Tetrahydro-4-hydroxy-6-methyl-2H-pyran-2-one";Acetoin acetate;Methyl levulinate;Pantolactone;	[M-H]$^-$	129.055718
^{13C6}Ketoleucine	[M-H]$^-$	135.075847
**Pyroglutamic acid**		
**Pyroglutamic acid**;Pyrrolidonecarboxylic acid;Pyrroline hydroxycarboxylic acid;N-Acryloylglycine;1-Pyrroline-4-hydroxy-2-carboxylate;5-Oxoprolinate;dimethadione	[M-H]$^-$	128.035317
^{13C5}Pyroglutamic acid	[M-H]$^-$	133.052091
**Glutathione**		
**Glutathione**	[M+H]$^+$<br>[M+Na]$^+$	308.091082<br>330.073025
^{13C5}Glutathione	[M+H]$^+$<br>[M+Na]$^+$	313.107857<br>335.089800
^{15N1}Glutathione	[M+H]$^+$<br>[M+Na]$^+$	309.088118<br>331.070060
**N-Acetyl-L-aspartic acid**		
**N-Acetyl-L-aspartic acid**;N-Formyl-L-glutamic acid;D-N-(Carboxyacetyl)alanine;2-Amino-3-oxoadipate;Berteroin	[M-H]$^-$	174.040796
^{13C3}N-Acetyl-L-aspartic acid	[M-H]$^-$	177.050861
^{13C4}N-Acetyl-L-aspartic acid	[M-H]$^-$	178.054215
^{15N1}N-Acetyl-L-aspartic acid	[M-H]$^-$	175.037831
**N-Formyl-L-glutamic acid**		
N-Acetyl-L-aspartic acid;**N-Formyl-L-glutamic acid**;D-N-(Carboxyacetyl)alanine;2-Amino-3-oxoadipate;Berteroin	[M-H]$^-$	174.040796
^{13C5}N-Formyl-L-glutamic acid	[M-H]$^-$	179.057570
^{15N1}N-Formyl-L-glutamic acid	[M-H]$^-$	175.037831
**D-Ribulose 5-phosphate/D-Ribose 5-phosphate**		
**D-Ribulose 5-phosphate**;Xylulose 5-phosphate;Ribose 1-phosphate;**D-Ribose 5-phosphate**;D-Xylulose 1-phosphate;D-Arabinose 5-phosphate;Beta-L-arabinose 1-phosphate	[M-H]$^-$	231.026430
^{13C5}D-Ribulose 5-phosphate/^{13C5}D-Ribose 5-phosphate	[M-H]$^-$	236.043205
**Sedoheptulose 7-phosphate**		

**D-Sedoheptulose 7-phosphate**;Sedoheptulose 1-phosphate	[M-H]$^-$	289.033007
^{13C5}D-Sedoheptulose 7-phosphate	[M-H]$^-$	294.049781
**Sedoheptulose 1,7-bisphosphate**		
**Sedoheptulose 1,7-bisphosphate**	[M-H]$^-$	368.999337
^{13C5}Sedoheptulose 1,7-bisphosphate	[M-H]$^-$	374.016111
**Valine**		
**L-Valine**;Betaine; Vaporole;N-Methyl-a-aminoisobutyric acid;5-Aminopentanoic acid;Norvaline;Amyl Nitrite	[M+H]$^+$<br>[M-H]$^-$	118.086255<br>116.071702
^{13C5}L-Valine	[M+H]$^+$<br>[M-H]$^-$	123.103029<br>121.088476
**alpha-Ketoisovaleric acid**		
**Alpha-ketoisovaleric acid**;Methylacetoacetic acid;Levulinic acid;2-Oxovaleric acid;2-Methylacetoacetic acid;Glutarate semialdehyde;Ethyl pyruvate;Acetoxyacetone	[M-H]$^-$	115.040068
^{13C5}Alpha-ketoisovaleric acid	[M-H]$^-$	120.056841
**(S)-3-Hydroxybutyric acid**		
**(S)-3-Hydroxybutyric acid**;2-Hydroxybutyric acid;(R)-3-Hydroxybutyric acid;(S)-3-Hydroxyisobutyric acid;(R)-3-Hydroxyisobutyric acid;3-Hydroxybutyric acid; 4-Hydroxybutyric acid;Alpha-Hydroxyisobutyric acid;Ethoxyacetic acid;2-Methyl-3-hydroxypropanoate]	[M-H]$^-$	103.040068
^{13C4}(S)-3-Hydroxybutyric acid	[M-H]$^-$	108.056842
**Propionylcarnitine**		
**Propionylcarnitine**	[M+H]$^+$	218.138685
^{13C3}Propionylcarnitine	[M+H]$^+$	221.148749
**Methylmalonic acid**		
**Methylmalonic acid**; Succinate;"Erythrono-1,4-lactone";Threonolactone	[M-H]$^-$	117.019332
^{13C3}Methylmalonic acid	[M-H]$^-$	120.029397
^{13C4}Methylmalonic acid	[M-H]$^-$	121.032752

Supplementary Figures

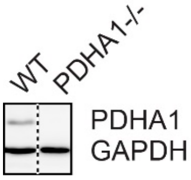

Figure S1. Western blot analysis of total lysates from wild type and PDHA1-/- HEK293T cells.

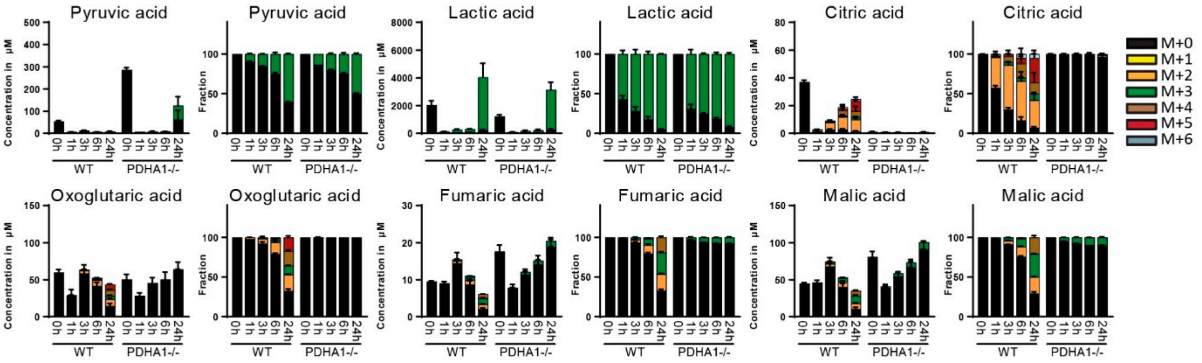

Figure S2. Targeted profiling of indicated metabolites in wild type and PDHA1 deficient HEK293T cells cultured in the presence of ^{13C_6}glucose for 0 h, 1 h, 3 h, 6 h and 24 hours.

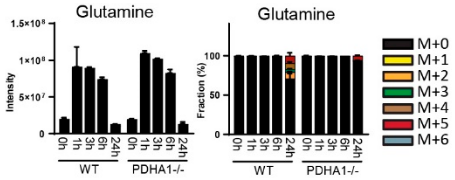

Figure S3. Total (labeled and unlabeled) intensity and fractional enrichment of glutamine in medium after 0 h, 1 h, 3 h, 6 h and 24 h incubation with ^{13C_6}glucose in wild type and PDHA1 deficient HEK293T cells. Isomeric compounds for each component are listed in Table S3.

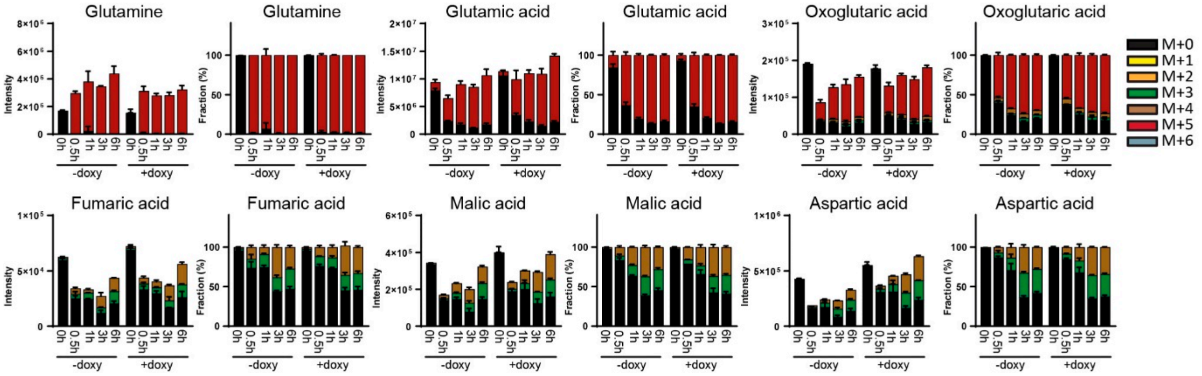

Figure S4. Metabolic consequences of overexpression of wild type GLS. Total (labeled and unlabeled) intensity and fraction of TCA cycle intermediates and aspartic acid after 0 h, 0.5 h, 1 h, 3 h and 6 h incubation with ^{13C_5}glutamine and with/without doxycycline in A549::pIND_GLS cells. Isomeric compounds for each component are listed in Table S3.
